# Arteriopathy in pediatric stroke: an underestimated clinical entity

**DOI:** 10.1590/0004-282X-ANP-2020-0105

**Published:** 2021-05-08

**Authors:** Ronaldo PIZZATTO, Lucas Lopes RESENDE, Carlos Felipe Teixeira LOBO, Yuri Costa Sarno NEVES, José Albino da PAZ, César Augusto Pinheiro Ferreira ALVES, Claudia da Costa LEITE, Leandro Tavares LUCATO

**Affiliations:** 1 Universidade de São Paulo, Faculdade de Medicina, Hospital das Clínicas, Instituto de Radiologia, São Paulo SP, Brazil. Universidade de São Paulo Universidade de São Paulo Faculdade de Medicina Hospital das Clínicas São Paulo SP Brazil; 2 Universidade de São Paulo, Faculdade de Medicina, Hospital das Clínicas, Instituto de Pediatria, São Paulo SP, Brazil. Universidade de São Paulo Universidade de São Paulo Faculdade de Medicina Hospital das Clínicas São Paulo SP Brazil

**Keywords:** Stroke, Chickenpox, Pediatric Emergency Medicine, Vasculitis, Central Nervous System, Moyamoya Disease, Acidente Vascular Cerebral, Varicela, Medicina de Emergência Pediátrica, Vasculite do Sistema Nervoso Central, Doença de Moyamoya

## Abstract

**Background:**

Pediatric arterial ischemic stroke (AIS), which was thought to be a rare disorder, is being increasingly recognized as an important cause of neurological morbidity, thanks to new advances in neuroimaging.

**Objective:**

The aim of this study was to review the main etiologies of stroke due to arteriopathy in children.

**Methods:**

Using a series of cases from our institution, we addressed its epidemiological aspects, physiopathology, imaging findings from CT, MR angiography, MR conventional sequences and MR DWI, and nuclear medicine findings.

**Results:**

Through discussion of the most recent classification for childhood AIS (Childhood AIS Standardized Classification and Diagnostic Evaluation, CASCADE), we propose a modified classification based on the anatomical site of disease, which includes vasculitis, varicella, arterial dissection, moyamoya, fibromuscular dysplasia, Takayasu's arteritis and genetic causes (such as ACTA-2 mutation, PHACE syndrome and ADA-2 deficiency). We have detailed each of these separately. **Conclusions:** Prompt recognition of AIS and thorough investigation for potential risk factors are crucial for a better outcome. In this scenario, neurovascular imaging plays an important role in diagnosing AIS and identifying children at high risk of recurrent stroke.

## RESUMO

**Introdução:**

O acidente vascular cerebral (AVC) pediátrico, considerado um distúrbio raro, está sendo cada vez mais reconhecido como importante causa de morbidade neurológica, graças aos novos avanços na neuroimagem.

**Objetivo:**

Revisar as principais etiologias do AVC por arteriopatia em crianças.

**Métodos:**

Utilizando-se de uma série de casos de nossa instituição, abordamos seus aspectos epidemiológicos, fisiopatológicos e de imagem na angiotomografia computadorizada e angiorressonância magnética, sequências convencionais e avançadas de ressonância magnética e medicina nuclear.

**Resultados:**

Com base na classificação mais recente de AVC na infância (Classificação Padronizada e Avaliação Diagnóstica do AVC na Infância - CASCADE) propusemos uma classificação modificada com base no local anatômico da doença, que inclui vasculite, varicela, dissecção arterial, Moyamoya, displasia fibromuscular, arterite de Takayasu e causas genéticas (como mutação ACTA-2, síndrome PHACE e deficiência de ADA-2), detalhando cada uma separadamente.

**Conclusões:**

O reconhecimento imediato do AVC na infância e a investigação minuciosa de possíveis fatores de risco são cruciais para um melhor resultado. Nesse cenário, a imagem neurovascular desempenha papel importante no diagnóstico de AVC e na identificação de crianças com alto risco de recorrência.

## INTRODUCTION

Pediatric arterial ischemic stroke (AIS) has long been thought of as a rare and benign condition. However, today it is being increasingly recognized as an important cause of neurological morbidity in children. Advances in noninvasive neuroimaging have led to more frequent diagnosing of this condition in children who could have been misdiagnosed with idiopathic cerebral palsy previously.

It used to be thought that children would have a good outcome after stroke. However, some studies have now shown higher rates of lifelong morbidity. Long-term neurological deficits have been observed in more than 75% of children after AIS and death has been a notable outcome (10%). The risk of recurrence has been estimated as 12%, one year after the stroke and 19%, five years after the stroke^1^^,^^2^^,^^3^^,^^4^.

Epidemiological studies have shown incidence rates of 1-6 cases per 100,000 individuals per year. Age has been seen to have a remarkable effect on incidence, such that children under the age of one year were found to be at a particularly higher risk (4-14 per 100,000 per year)^5^^,^^6^.

The aim of the present study was to review the main etiologies of childhood stroke and its image presentation.

## IMAGING METHODS

Because of the high incidence of stroke mimics in childhood, a diagnosis of AIS requires confirmation of an ischemic lesion. Thus, MRI with DWI should be performed, given its higher sensitivity for acute ischemia^7^.

Magnetic resonance angiography (MRA) of the cervical and intracranial arteries should be performed when vasculopathy is suspected. Its accuracy is similar to that of conventional angiography for vasculopathy, except for small vessel involvement, with the advantages of no radiation exposure and no need for venipuncture. Nevertheless, conventional angiography remains the gold standard for diagnosing cerebral arteriopathy^8^^,^^9^^,^^10^.

CT angiography also plays a role in investigating AIS. Although there no studies specific for pediatric population, it might give rise to fewer false-positive findings for stenosis and occlusion in the posterior circulation than MRI, when there is minimal flow in this region^11^.

In the present study, we evaluated images taken at our institution between 2014 and 2016. Computed tomography (CT) images were taken using multislice scanners with at least 16 channels (16-slice scanners). CT angiography of the intracranial and cervical vessels was performed with 1 mm-collimation and subsequent 3D-volume rendering. Magnetic resonance imaging (MRI) was obtained using 1.5 Tesla scanners, and we performed MR angiography on all patients. The MRI protocols included T1, T2, FLAIR, SWI and DWI without gadolinium-based contrast, and 3D-TOF and 3D-T1 with intravenous gadolinium-based contrast. Experienced neuroradiologists evaluated all images.

## ARTERIOPATHY AND STROKE

The risk factors for stroke in children are markedly different from those in adults. They also vary substantially between the perinatal period and childhood^6^. Perinatal AIS has multifactorial causes due to the relatively hypercoagulable nature of pregnancy itself and the complex interaction between the maternal and the fetal circulation^6^. In childhood, arteriopathy is the most important risk factor for stroke and it is found in up to half of all children with AIS. Cerebral arteriopathy on neuroimaging has also been associated with recurrent stroke^3^^,^^12^^,^^13^.

There is evidence for an arteriopathy syndrome involving a primary inflammatory mechanism unique to the cerebral arteries. Pathology-based models have proven that small-vessel central nervous system vasculitis is inflammatory-mediated, while large-vessel arteriopathy presents many features that suggest inflammatory mechanisms such as better outcomes with corticosteroid treatment^14^.

## CLASSIFICATION OF CHILDHOOD ARTERIAL ISCHEMIC STROKE

In order to provide a standardized language for describing the types of strokes that are often encountered, the Childhood AIS Standardized Classification and Diagnostic Evaluation (CASCADE) criteria were created. The primary CASCADE classification is based on the anatomical site of disease, including the heart, great vessels of the neck and intracranial vessels. The secondary classification includes genetic causes for arteriopathy and also hemoglobinopathy and infections as stroke types^15^^,^^16^.

With the aim of including only cases with arteriopathy, we modified this classification to exclude the cardioembolic causes and some secondary causes such as infection, hematological causes, inflammatory causes, toxins and vasospasm. This modified classification relates to acute strokes, i.e. situations in which the clinical history and imaging are obtained within one month after the onset of symptoms. It is shown in Table 1.

**Table 1.** Modified Classification of Childhood Arterial Ischemic Stroke. This modified classification only includes cases with arteriopathy and with an acute presentation, which occurs when the clinical history and imaging are obtained within one month after the onset of symptoms*.

Modified Classification of Childhood Arterial Ischemic Stroke
Small-Vessel Arteriopathy Primary vasculitis Secondary vasculitis
Unilateral Focal Cerebral Arteriopathy Varicella Arterial dissection
Bilateral Cerebral Arteriopathy Moyamoya
Aortic / Cervical Arteriopathy Cervical dissection Fibromuscular dysplasia Takayasu's
Genetics ACTA-2 mutation PHACE syndrome

*Modified from Bernard et al.^16^.

## SMALL VESSEL ARTERIOPATHY

### Vasculitis

Central nervous system vasculitis is a less frequent cause of childhood AIS. Its pathophysiology is based on irregular vascular stenosis that results in both deep and superficial sites of ischemia, not respecting any major vascular area^17^^,^^18^.

Small-vessel cerebral vasculitis can be subdivided into primary, affecting only the central nervous system (e.g. small-vessel childhood primary angiitis of the central nervous system); and secondary, associated with other systemic disorders such as collagen vascular disease or septic meningitis^19^.

**Figure 1.**
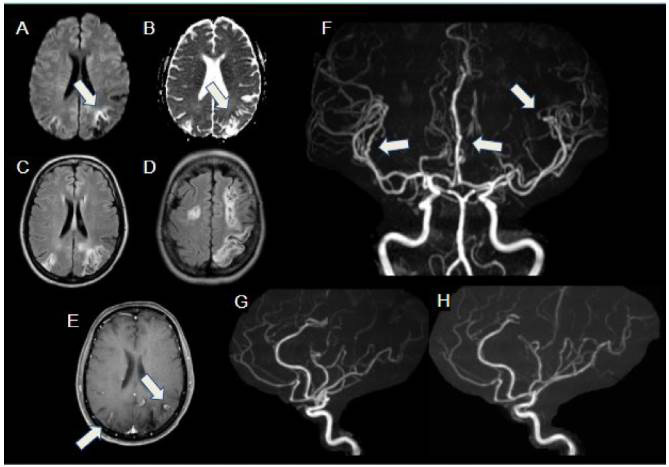
Systemic lupus erythematosus vasculitis. Multiple infarctions affecting different vascular regions, in various stages of healing in a 14-year-old female patient presenting with right lower limb weakness. Hyperintensity areas on DWI (A) with corresponding low-signal ADC map (B) show restricted diffusion in the left parietal region, in the anterior border of a previous infarct, compatible with acute stroke (arrows). Axial FLAIR images (C and D) show multiple cortical-subcortical areas of infarction in different vascular regions of both cerebral hemispheres, predominantly on the left. Axial T1 C+ FS (E) shows cortical areas of contrast enhancement in parietal regions, compatible with subacute / chronic infarctions (arrows). MRA images (F, G and H) depict multiple irregularities and stenosis of multiple distal artery branches (arrows), compatible with vasculitis.

Secondary CNS vasculitis may be the presenting symptom in childhood rheumatic diseases or may develop over the course of illness. CNS vasculitis is seen in children with the following: systemic lupus erythematosus (Figure 1); anti-neutrophil cytoplasmic antibody-associated systemic vasculitis, including granulomatosis with polyangiitis (previously known as Wegener granulomatosis) and microscopic polyangiitis (MPA); polyarteritis nodosa (PAN); and Takayasu arteritis. The treatments for secondary CNS vasculitis in rheumatic diseases commonly include high-dose corticosteroids and cyclophosphamide^20^^,^^21^.

**Figure 2.**
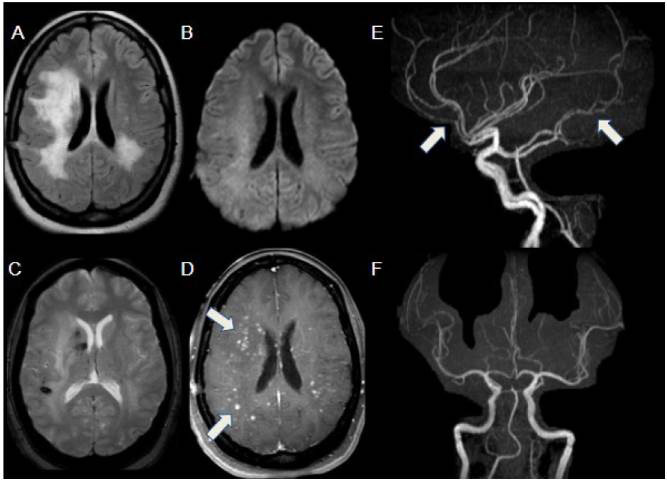
Primary angiitis of the central nervous system, in 17-year-old female with progressive unexplained neurological deficits. Axial FLAIR image (A) shows an extensive area of abnormal signal in the right frontoparietal white matter and left peritrigonal white matter, without restricted diffusion (B). SWI (C) depicts foci of magnetic susceptibility in the right caudate and subcortical white matter, compatible with microhemorrhages. After administration of gadolinium (D), multiple bilateral parenchymal nodular foci of enhancement are evident in the areas of abnormal FLAIR signal, with perivascular distribution (arrows). MRA images (E and F) show irregularities in multiple distal arterial branches (arrows), consistent with vasculitis.

Conventional angiography is the best imaging method and it is safe in pediatric populations. In small-vessel childhood primary angiitis of the central nervous system, alternating areas of stenosis and dilatation can be seen in distal arterial beds (Figure 2)^22^.

Abnormalities seen on magnetic resonance imaging are often present even in angiogram-negative cases. These abnormalities may include asymmetric supratentorial and anterior circulation lesions of the white matter or deep gray matter structures. Enhancement is also occasionally observed, while hemorrhagic lesions are barely seen. Areas of restriction on diffusion weighed images are reported in up to 60% of the patients^17^^,^^23^.

The predictors of progressive vasculitis that are seen on imaging include multifocal, bilateral and gray matter lesions, along with multiple, bilateral or distal vessel stenosis^23^.

## UNILATERAL FOCAL CEREBRAL ARTERIOPATHY

### Varicella

**Figure 3.**
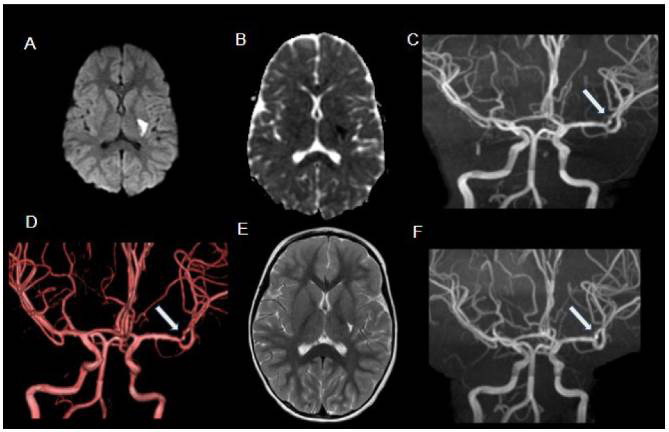
Varicella. Acute stroke in a 3-year-old female patient presenting with lower-limb weakness, weeks after chickenpox. DWI (A) and corresponding ADC map (B) show restricted diffusion in the posterior left putamen and internal capsule. MRA (C and D) depicts irregularities in the M1-M2 segments of the left middle cerebral artery (arrows). Follow-up MRI after 5 months demonstrates residual findings (E, T2WI) and partial resolution of the vascular irregularities (F, MRA, arrow).

Varicella vasculopathy (Figure 3) accounts for 30% of all AIS in children and often occurs in weeks to months after cutaneous manifestation of zoster or varicella^24^. Children with stroke were 18 times more likely to have had chicken pox in the previous 9 months than healthy controls^7^.

The typical angiographic changes include segmental constriction/stenosis, often with post-stenotic dilatation, or occlusion^25^. A negative angiogram does not rule out the diagnosis, because disease in small arteries is not detectable as readily as in large arteries. Overall, involvement of large and small arteries is found more often at the same time in a single patient than is pure small-artery or pure large-artery disease. This last one is even reported less often^26^.

Cortical and deep white matter lesions are found, mostly ischemic, but hemorrhagic lesions also occur. Some are enhanced on MRI with contrast, thus indicating breakdown of the blood-brain barrier^26^. The diagnosis of varicella vasculopathy is made when CSF anti-VZV IgG antibodies or VZV DNA are found^27^.

### Arterial dissection

**Figure 4.**
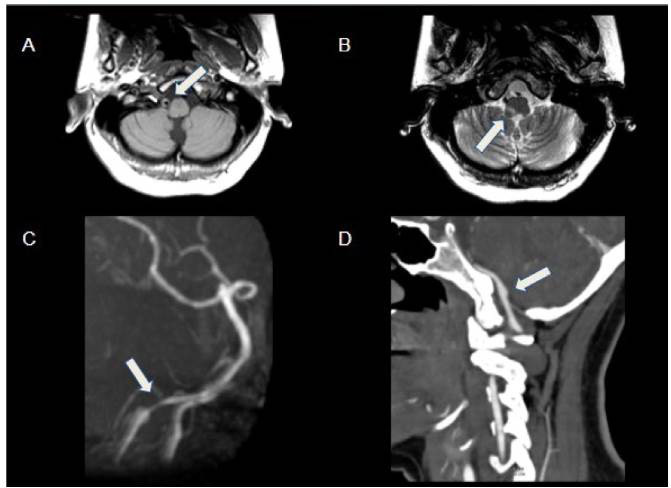
Arterial dissection in 16-year-old female with acute onset of Wallenberg syndrome. Axial T1WI (A) shows high-signal eccentric thickening in the right vertebral artery, compatible with mural hematoma (arrow). T2WI (B) depicts infarcted area in the right dorsolateral portion of the medulla oblongata (arrow). MRA (C) highlights tapering of the V4 segment of the right vertebral artery (arrow), suggestive of dissection. Follow-up CTA (D) shows right V4 dissecting a pseudoaneurysm (arrow).

Arterial dissection (AD) of the craniocervical or intracranial vessels (Figure 4) is an underrecognized cause of arteriopathy in children, particularly when relying only on MR angiography for diagnosis^28^.

There are several differences between adult and childhood arterial dissections. Intracranial dissection is more common in children than in adults, and it often occurs without the preceding history of trauma that is typically expected with extracranial dissection. Predominant involvement of the anterior circulation (60%) was observed in some studies, in most cases with no identified predisposing factor, and this was classified as spontaneous. In cases of involvement of the posterior circulation, males predominate and these cases are more related to histories of trauma^29^^,^^30^.

Symptoms of AIS or transient ischemic attack are usually presented. Pain is not a remarkable feature. Headache is reported in only half of the patients and neck pain is barely reported. This contrasts with adults, in whom pain is often noted to be the most common presenting feature^31^.

Conventional angiography (CA) is still widely considered to be the gold standard for diagnosing of adult and childhood craniocervical arterial dissection (CCAD), but the risks of this technique may outweigh its benefits in many clinical scenarios. CA depicts intraluminal findings of CCAD with very high spatial resolution, through direct intra-arterial injection of contrast. The specific findings from arterial dissection are intimal flap, pseudoaneurysm, mural hematoma and arterial occlusion^28^.

In most centers, MRI/MRA has become the first-line imaging modality for patients with suspected dissection^32^^,^^33^. MRI/MRA is noninvasive, uses no radiation, and enables simultaneous imaging for dissection and stroke. The arterial luminal findings of CCAD using either time-of-flight (TOF) MRA or contrast-enhanced MRA are similar to the findings of CA in both adults and children, including arterial stenosis, intimal flap, dissecting aneurysm or occlusion. Although CA is the gold standard for making the diagnosis, one advantage of MRI/MRA (TOF or contrast-enhanced) over CA is the ability to directly visualize intramural hematomas through T1 or T2 fat-saturated imaging, as a crescentic hyperintensity along the vessel wall^34^.

There is a lack of studies regarding the true prevalence and importance of connective tissue disorders such as Marfan, Ehlers-Danlos and Loeys-Dietz syndromes. Nonetheless, these disorders are not negligible, especially in patients with evidence of multiple dissections^35^. The long-term complications following dissection can include development of pseudoaneurysms, and these complications can be monitored by means of MRI/MRA, CT/CTA and cervicocranial catheter angiograms^19^.

## BILATERAL CEREBRAL ARTERIOPATHY

### Moyamoya

Moyamoya is a progressive noninflammatory arteriopathy involving the distal carotid artery that was first described in Japan, where it remains most prevalent. The term was originally coined to describe the hazy appearance of the network of basal collateral vessels that are formed as the result of progressive stenosis of the distal carotid arteries^36^. Pathologically, it is characterized by fibrocellular intimal thickening, possibly due to a constrictive remodeling process^37^.

It can be sporadic or hereditary. In contrast to idiopathic moyamoya disease, moyamoya syndrome refers to similar angiographic findings that occur in the context of other underlying diseases, including trisomy 21, neurofibromatosis type 1 and sickle cell anemia. Susceptibility loci are beginning to be identified, such as the RFN213 gene^37^.

**Figure 5.**
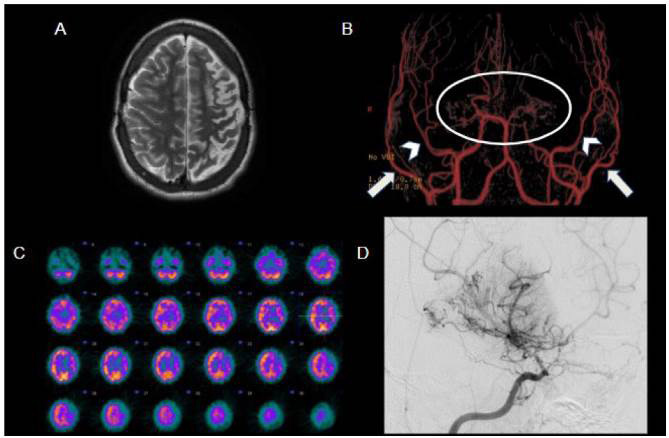
Moyamoya syndrome secondary to sickle cell disease. 18-year-old male patient with sickle cell disease and history of multiple cerebrovascular events, who underwent bilateral encephalo-duro-arterial synangiosis (EDAS). Axial T2WI (A) shows a large left frontoparietal area of encephalomalacia. MRA (B) depicts progressive tapering of the distal internal carotid arteries, culminating in distal subocclusion/occlusion (circle), with extensive collateral vessel network deriving from external carotid arteries; the dilated branches of these arteries, especially the medial meningeal arteries (arrowheads) and superficial temporal arteries (arrows), are related to EDAS. SPECT (C) demonstrates reduced perfusion in the left cerebral hemisphere, while the right hemisphere is relatively preserved, possibly due to EDAS. Digital subtraction angiography (D) on another sickle cell patient (13 years old) outlines the classical angiographic pattern of moyamoya syndrome (internal carotid artery stenosis with compensatory hypertrophy of lenticulostriate arteries).

The diagnosis is made by means of arteriography (gold standard), CTA and MRA, with demonstration of typical findings of progressive steno-occlusive arteriopathy that typically involves the distal internal carotid artery (ICA) and proximal MCAs or ACAs bilaterally (Figure 5). It can also can affect the posterior circulation, although this is much less common^38^.

Understanding this disease is important because its treatment is unique in the context of other arteriopathies, given that it relies on surgical revascularization^38^. Although arteriography is the gold standard for postoperative evaluation in moyamoya disease, some studies have reported that MR perfusion and SPECT are important tools for depicting hemodynamic status after revascularization surgery^39^.

## AORTIC/CERVICAL ARTERIOPATHY

### Fibromuscular dysplasia

Fibromuscular dysplasia is a noninflammatory arteriopathy. It is only rarely seen in childhood and is associated with ischemic and hemorrhagic AIS^40^.

**Figure 6.**
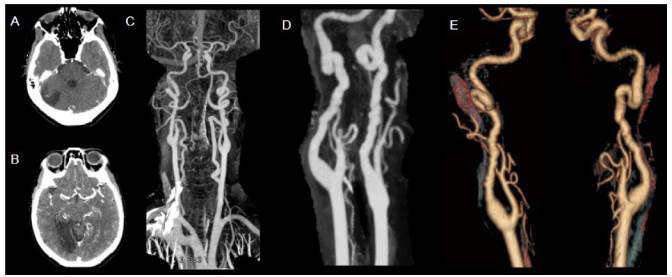
Fibromuscular dysplasia in 60-year-old patient under investigation regarding acute reduction of visual acuity. Axial CT after contrast administration shows infarcted areas in posterior circulation (A and B). CTA with MIP (C and D) and 3D reconstruction (E) show bilateral irregularity and tortuosity of the internal carotid arteries, forming vascular loops in a classic string-of-beads fashion. This classical imaging presentation is not commonly seen in children, who usually have less specific arterial changes that are described as focal or segmental stenosis or occlusions.

The cervical vasculature is most frequently involved, classically described as having alternating areas of vascular constriction and dilatation (*string-of-beads* angiography pattern), which is specific, although not sensitive (Figure 6)^41^^,^^42^.

Most children with pathologically proven fibromuscular dysplasia did not have string-of-beads angiography pattern. They usually have less specific arterial changes, described as focal or segmental stenosis or occlusions^40^.

Making a definitive diagnosis of fibromuscular dysplasia is difficult. It is usually made by excluding other causes of arteriopathy. The gold standard is histopathological analysis. Serial vascular imaging studies are also probably required so that disease activity over time can be followed up well, and these play a role in the differential diagnosis^40^.

### Takayasu’s arteritis

Takayasu’s arteritis (TA) is a chronic, potentially progressive, granulomatous inflammation of the aorta and its main branches and it is the most frequent pediatric large-vessel vasculitis. The prevalence of TA in adults is reported to be one in one million, but pediatric incidence data for TA are not yet available^43^. The majority of cases among children are in females, and they are diagnosed during adolescence, at a mean age of 13 years^44^.

The most commonly involved vessels in children are the aorta and the renal, subclavian and carotid arteries (Figure 4)^44^. The typical manifestations of TA comprise diminished or absent pulses associated with claudication, vascular bruits or hypertension^43^.

The diagnosis in children is based on the European League Against Rheumatism (EULAR)/PRES criteria: angiographic abnormalities plus decreased peripheral artery pulse(s) and/or claudication of extremities OR a blood pressure difference > 10 mmHg OR bruits over the aorta and/or its major branches OR hypertension^45^.

In ultrasound evaluations, the following can be observed: long segments of smooth, homogeneous and concentric wall thickening (“macaroni” sign) and hemodynamic consequences of changes in the vessel lumen. Ultrasound assessment of multiple vessels is challenging and time-consuming^46^.

**Figure 7.**
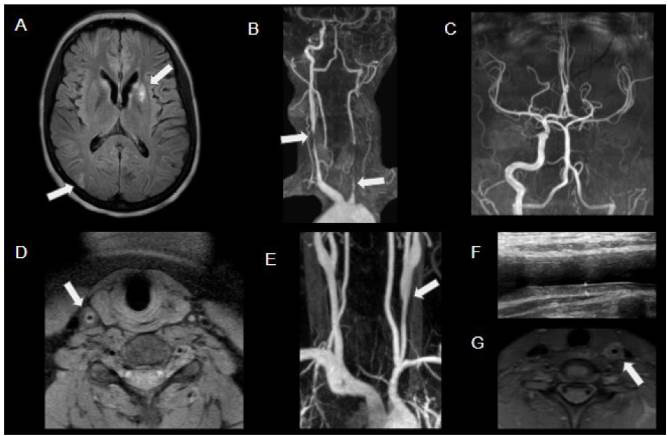
Takayasu’s arteritis in a patient with acute stroke in the left lenticulostriate region. Axial FLAIR (A) shows infarcted areas in the left nucleocapsular region and in the right posterior border zone (arrows). MRA imaging shows stenosis of the right common carotid artery, occlusion of the left common carotid artery (arrows), and also from both vertebral arteries, with distal filling by collaterals (B and C). Axial T1WI (D) shows circumferential thickening of the common right carotid artery (arrow). MRA (E) on another female teenager with Takayasu’s arteritis showing left common carotid stenosis (arrow). There was corresponding wall thickening on grayscale ultrasound (calipers in F) and marked wall thickening and enhancement on MR T1WI C+ FS (arrow in G).

CT and MRI are noninvasive procedures in which early vessel wall changes before luminal impairment can be observed. They may also play a role in differentiation between stenosis caused by inflammation and fibrosis, and also allow simultaneous assessment of the pulmonary arteries. CT and MRI angiograms help by depicting the luminal changes and collateral formation (Figure 7)^46^.

Digital subtraction angiogram (DSA) still plays an important role in guiding interventions such as angioplasty and stent placement^47^.

## GENETICS

### Acta-2 mutation

**Figure 8.**
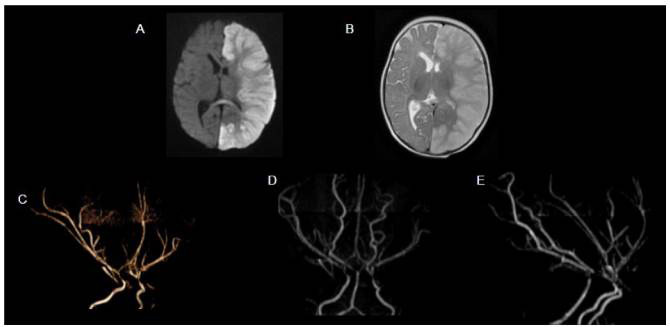
ACTA-2 mutation in 10-month-old female infant presenting with extensive left cerebral hemisphere infarct. DWI (A) shows a large hemispherical area of restricted diffusion. Axial T2WI (B) shows corresponding diffuse high signal intensity and mass effect. MRA with 3D rendering (C, D and E) demonstrates rectification of the intracranial arteries (loss of normal curvature) and tapering of distal internal carotid arteries.

Heterozygous ACTA-2 gene mutations cause smooth muscle impairment in many vessels, including the cerebral arteries, thoracic aorta and dermal and cardiac arteries (Figure 8)^48^.

Cerebral arteriopathy is characterized by the following: dilated extradural arteries; straightening and narrowing of the intradural arteries; large arterial occlusions without the lenticulostriate collaterals seen in Moyamoya disease; and distal small artery aneurysms and corkscrewing, more prominently in the posterior circulation^49^.

Early diagnosis is essential for optimal management of these patients. MRI with MRA is the best method for making the diagnosis and for follow-up. CT may help in the acute phase in patients presenting with stroke, while DSA should be performed only when a therapeutic revascularization approach is intended^50^.

Genetic testing is essential for evaluating the patient’s family, in order to provide an accurate prognosis and genetic counseling. The ACTA-2 mutation has invariably been associated with a poor prognosis, with high risk of death in infancy^50^.

### PHACE syndrome

**Figure 9.**
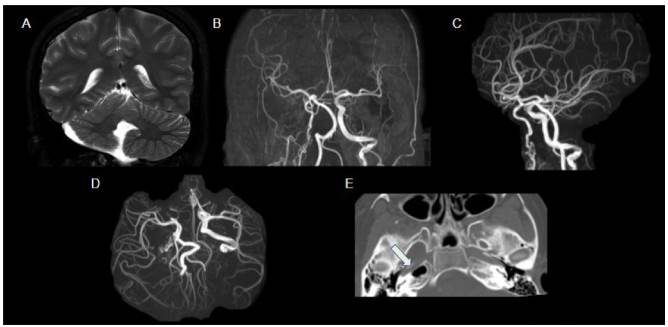
PHACE syndrome in 12-year-old female with a right-face hemangioma. Coronal T2WI (A) shows asymmetry of cerebellar hemispheres with volumetric reduction of the right hemisphere and hypoplastic vermis. MRA (B, C and D) shows thinning of the right internal carotid artery and multiple tortuous and irregular ipsilateral vessels (rete mirabile). CT of the skull base (E) depicts narrowing of the right internal carotid canal (arrow), thus signaling hypoplasia.

PHACE syndrome comprises a spectrum of anomalies, including posterior fossa malformations, hemangiomas, arterial anomalies, cardiac defects, eye anomalies and sternal cleft or supraumbilical raphe (Figure 9). For a definitive diagnosis of PHACE syndrome, the patient needs to present a infantile hemangioma on the face or scalp > 5 cm^51^.

Cervical and intracranial arteriopathy has been reported to be the most common extracutaneous abnormality in this disorder. It was found to occur with an estimated prevalence of 84%, in a national registry of patients^52^.

The range of arterial anomalies includes agenesis, luminal narrowing, dolichoectasia, persistence of primitive embryonic arteries, aneurysms and, less commonly, progressive postnatal narrowing, moyamoya-like collaterals and thrombosis^53^^,^^54^^,^^55^^,^^56^^,^^57^^,^^58^.

The vessels are almost always found ipsilaterally in relation to the hemangioma. The vessel most commonly involved is the internal carotid artery or its early embryological branches^58^. Structural brain abnormalities are frequently associated with PHACE syndrome, and the posterior fossa is the most common location. Unilateral cerebellar hypoplasia has been reported to be the most common abnormality. Supra-tentorial findings include callosal dysgenesis and malformations of cortical development. Unilateral lesions are also usually ipsilateral to cutaneous hemangioma^58^.

### ADA-2 deficiency

Deficiency of adenosine deaminase 2 (ADA-2) is a recently described autoinflammatory disease caused by loss-of-function homozygous or compound heterozygous mutations in the CECR1 (Cat Eye Syndrome Chromosome Region 1) gene. ADA-2 deficiency may compromise endothelial integrity while polarizing macrophage and monocyte subsets toward proinflammatory cells, thus establishing a vicious circle of vasculopathy and inflammation.

**Figure 10.**
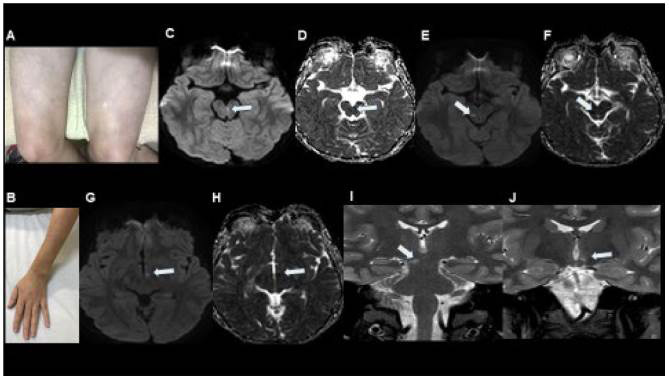
ADA-2 deficiency in 13-year-old boy with livedo reticularis (A, B) and sudden onset of left ophthalmoplegia. DWI and ADC mapping (C, D) shows restricted diffusion in the left paramedian portion of the mesencephalon in the projection of the left III cranial nerve fibers (arrows), compatible with acute stroke. One year later, this boy presented with another acute symptom, this time consisting of paresthesia and loss of taste in the left portion of the tongue. DWI and ADC mapping (E, F, G, H) showed restricted diffusion in the right paramedian portion of the mesencephalon and in the left hypothalamus (arrows), both compatible with acute stroke. Coronal T2-weighted images (I, J) confirmed these findings (arrows). Whole-exome sequencing was performed and showed heterozygosity for missense mutations in CECR1, thus encoding ADA-2.

ADA-2 deficiency is characterized by early-onset vasculopathy with the clinical and histopathological features of polyarteritis nodosa (PAN), associated with hemorrhagic and ischemic strokes (Figure 10). Hypogammaglobulinemia with reduction of memory and terminally differentiated B cells and plasma cells may be present. A severe clinical picture dominated by cytopenia and lymphoproliferation has also been described. Although the onset of this disease is commonly at pediatric ages, some patients with onset in adulthood have been described as well^59^.

It is also possible that ADA-2 deficiency accounts for some patients with Sneddon’s syndrome, a poorly understood disorder that is most common in middle-age women and which is characterized by livedoid rash and stroke, with antiphospholipid antibodies present in some of the patients^60^.

Childhood AIS is an important cause of childhood morbidity. Prompt recognition of AIS and thorough investigation for potential risk factors are crucial for a better outcome. Vascular imaging is extremely important for identifying children at high risk of recurrent stroke, and it is imperative to know the imaging presentation of the main causes of arteriopathy in this group. Interpretation of these imaging presentations is facilitated through the modified CASCADE classification proposed in this study.

Improvements in care systems and standardized care pathways, larger-scale studies on treatment strategies and novel technologies in neuroimaging and neurorehabilitation will lead to better understanding of the pathophysiology of pediatric AIS and enable achievement of better outcomes in this population.
